# Rapid Detection of *Staphylococcus aureus* in Milk Samples by DNA Nanodendrimer-Based Fluorescent Biosensor

**DOI:** 10.3390/bios15080527

**Published:** 2025-08-12

**Authors:** Mukaddas Mijit, Dongxia Pan, Hui Wang, Chaoqun Sun, Liang Yang

**Affiliations:** 1 State Key Laboratory of Animal Nutrition and Feeding, Institute of Animal Science, Chinese Academy of Agricultural Sciences, Beijing 100193, China; 82101225447@caas.cn (M.M.); 82101222382@caas.cn (D.P.); 2 Animal Science and Technology College, Guangxi University, Nanning 530004, China; 82101235459@caas.cn

**Keywords:** biosensor, DNA nanodendrimer, DNAzyme, fluorescence amplification, *Staphylococcus aureus*, milk safety

## Abstract

*Staphylococcus aureus* is the primary pathogen responsible for mastitis in dairy cows and foodborne illnesses, posing a significant threat to public health and food safety. Here, we developed an enhanced sensor based on solid-phase separation using gold-magnetic nanoparticles (Au@Fe_3_O_4_) and signal amplification via dendritic DNA nanostructures. The substrate chain was specifically immobilized using thiol–gold coordination, and a three-dimensional dendritic structure was constructed through sequential hybridization of DNAzymes, L chains, and Y chains, resulting in a 2.8-fold increase in initial fluorescence intensity. Upon specific cleavage of the substrate chain at the rA site by *S. aureus* DNA, the complex dissociates, resulting in fluorescence intensity decay. The fluorescence intensity is negatively correlated with the concentration of *Staphylococcus aureus*. After optimization, the biosensor maintains a detection limit of 1 CFU/mL within 3 min, with a linear range extended to 1–10^7^ CFU/mL (R^2^ = 0.998) and recovery rates of 85.6–102.1%, significantly enhancing resistance to matrix interference. This provides an innovative solution for rapid on-site detection of foodborne pathogens.

## 1. Introduction

*Staphylococcus aureus*, as a significant pathogen of global foodborne illnesses, has been widely validated as a public health threat. Data from the USA. Center for Disease Control and Prevention show that bacterial infections caused by this pathogen rank second only to *Escherichia coli*, consistently maintaining its position as the second most prevalent foodborne pathogenic bacterium [1]. Its pathogenicity is mainly due to the potent emetic properties of heat-resistant enterotoxins (SEs), which can remain active at 100 °C for up to 30 min and are difficult to inactivate by conventional pasteurization [2]. It is of concern that dairy mastitis is a key risk node in the dairy contamination chain: more than 50% of dairy mastitis cases are directly caused by *Staphylococcus aureus*, and the detection rate of this bacterium in the milk of diseased cows is as high as 94–100% [3], which makes dairy products high-risk vectors for SE contamination. Epidemiological studies have shown that in the case of *Staphylococcus aureus* contamination associated with dairy products, even if the initial bacterial load is less than 10 CFU/mL, the exponential accumulation of the toxin during the storage period can still exceed the threshold for human intoxication (20–100 ng/g), highlighting the urgency of controlling the contamination at the source [4]. Therefore, effective, precise, and rapid detection of *Staphylococcus aureus* is important for food safety.

The traditional detection methods for *S. aureus* mainly rely on bacterial isolation and culture, smear microscopy, biochemical tests, animal infection tests, and serotyping, which have problems such as time-consuming detection, complicated operation, and the need for professional personnel [5,6]. In the last decade, fluorescent biosensors have an important potential as a promising spectroscopic analysis technique in the field of biomolecular detection [7]. For example, Gao et al. [8] designed a novel dual-mode biosensor that combines the CRISPR/Cas12a system with an enzyme-free isothermal amplification method, with a detection limit as low as 5.7 CFU/mL for *Staphylococcus aureus* and as low as 133.7 CFU/mL in fluorescence mode, that demonstrated excellent accuracy, stability and sensitivity. Neha et al. [9] have developed a biosensor for the conjugation of *Staphylococcus aureus* phage with the water-dispersible and environmentally stable metal organic framework (MOF) NH_2_-MIL-53 (Fe). The affixation of MOF to phage has been achieved by using glutaraldehyde as a cross-linking agent. The proposed MOF phage biosensor achieves a highly sensitive detection of *Staphylococcus aureus* in both synthetic and real samples based on the photoluminescence burst phenomenon with a detection limit of 31 CFU/mL.

Recent advancements in nanotechnology have significantly enhanced the detection performance of fluorescent biosensors in food samples. Fluorescent biosensors that use novel bio-sensitive materials and nanomaterials (such as quantum dots, graphene oxide, and metal nanoparticles) have achieved ultra-sensitive detection of pathogens through signal amplification and enhanced surface reactivity [10,11,12]. For example, CRISPR-Cas-integrated biosensors can achieve attomole-level sensitivity but require complex nucleic acid extraction [13], while aptamer-metal organic framework (MOF) conjugates, though capable of rapid response, are subject to matrix interference in dairy products [14]. Despite these advancements, key challenges remain: (1) most platforms require time-consuming sample pretreatment for complex matrices like milk; (2) multiplex detection is limited by non-specific binding; and (3) portable devices with real-time output capabilities are scarce. To address these issues, the latest research findings regarding DNA-sensitive materials and novel nanoscale Au@Fe_3_O_4_ nanoparticles can provide new solutions.

Functional Nucleic Acids (FNAs) are a class of nucleic acid molecules constructed through rational design or directed evolutionary strategies, which break through the limitations of traditional nucleic acids as carriers of genetic information, and exhibit diverse biological functions such as molecular recognition, catalytic activity, and dynamic regulation [15,16]. Its core strengths derive from the inherent structural programmability of nucleic acid molecules and compatibility with chemical modifications [17]. Based on their functional properties, FNAs can be classified into three categories: molecular recognition type represented by aptamers (Aptamer), which bind targets with high affinity through three-dimensional structures; catalytically active type centered on DNAzyme/RNAzyme, which mimic natural enzymes to achieve substrate cleavage or synthesis reactions; and dynamically regulated type based on DNA nanomachines, which perform logical operations through conformational changes [18,19]. Compared with protein-based tools, FNAs are thermally stable, low-cost, and have high batch-to-batch consistency. They have already shown transformative potential in fields such as biosensing, targeted therapeutics, and synthetic biology [20,21,22].

Deoxyribonucleases (DNAzymes) are a class of functional DNA molecules with catalytic activity, capable of specifically catalyzing a wide range of chemical reactions, including phosphodiester bond breaking and peroxide decomposition [23,24]. The discovery of DNAzymes breaks through the boundary of the traditional enzyme science that the catalytic function is only mediated by proteins or RNA. Compared to traditional proteases, DNAzymes are composed of single- or double-stranded DNA and combine the programmability, sequence specificity, and excellent chemical stability of nucleic acid molecules, providing novel tools for biocatalysis [25,26]. Currently, two classes of typical DNAzymes show important applications: (1) G-quadruplex/hemin complexes with peroxidase-like activity, which can be used to construct colorimetric sensors through catalytic chromogenic reactions [27]; and (2) RNA-cleaving DNAzymes, capable of recognizing specific RNA sequences and catalyzing their cleavage through base complementary pairing, show targeted regulatory potential in gene silencing therapy [28,29]. In the field of analytical testing, the substrate recognition-signal transduction coupling property of DNAzymes has been widely used to construct high-sensitivity biosensors for the precise detection of heavy metal ions, pathogen nucleic acids, and disease markers [30,31,32]. In addition, DNAzymes, as molecular tools, provide a new research paradigm for revealing the catalytic mechanism of nucleic acids and developing dynamic nanodevices, which continue to promote cross-innovation between chemical biology and synthetic biology [33].

Biosensing technology based on the aggregation effect of nanomaterials has become a research hotspot in the fields of biomedical diagnostics and environmental monitoring due to its advantages of high sensitivity, fast response, and visualized output [34]. However, traditional magnetic bead sensors face two major technical bottlenecks: firstly, the non-specific aggregation of nanoparticles is prone to lead to false-positive signals, especially in complex matrices where interference is significant; secondly, the cumbersome process of functionalization and modification of the magnetic bead surface severely limits its applicability to on-site testing [35]. To break through these limitations, researchers are working to develop composite magnetic bead carriers; material properties can be significantly enhanced by encapsulating polymers, silica, or precious metals [36,37,38]. Among them, gold nanoparticle-modified Au@Fe_3_O_4_ nanoparticles (AuNPs/MBs) stand out due to their unique interfacial properties—the high specific surface area and chemical stability of AuNPs not only prolongs the sensor shelf-life, but also enables high-density targeted immobilization of aptamers through Au–S covalent bonding [39,40]. This precise modification strategy not only enhances target capture efficiency, but also synergizes with antibodies and fluorescent markers to build multimodal sensing interfaces [41,42]. These advances have laid the material foundation for the development of the next generation of marker-less, all-in-one field inspection equipment.

To address these challenges, we developed a fluorescent biosensor integrating a Y-shaped DNA dendrimer and DNAzyme-catalyzed signal switching for ultrasensitive *S. aureus* detection in milk. The biosensor features three key innovations: (1) a Y-shaped DNA dendrimer with a branched architecture enabling multistage signal amplification to enhance sensitivity; (2) Au@Fe_3_O_4_ nanoparticles that streamline probe immobilization via carboxyl-Au bonding, optimizing target capture efficiency while minimizing nonspecific adsorption; and (3) a DNAzyme-mediated catalytic cleavage mechanism, where *S. aureus* activates DNAzyme to cleave fluorescent dendrimers from Au@Fe_3_O_4_ nanoparticles, triggering a proportional reduction in fluorescence intensity.

## 2. Materials and Methods

### 2.1. Chemicals and Apparatus

Chemicals: Au@Fe_3_O_4_ nanoparticles (Au@Fe_3_O_4_, 80 nm diameter) were purchased from Sigma-Aldrich (St. Louis, MO, USA); DNA sequences (Substrate chain, DNAzyme, L1, L2, Y1, Y2, Y3) with 5′-SH and FAM fluorescence modifications were synthesized by Sangun Biotech Co., Ltd. (Shanghai, China) as shown in Table 1; *Staphylococcus aureus* (ATCC 25923), *Escherichia coli*, *Salmonella typhimurium*, *Bacillus subtilis*, and *Streptococcus lactis* strains were obtained from Beijing Xiecheng Biotechnology Co., Ltd. (Beijing, China).

Buffers and solutions: Phosphate-buffered saline (PBS, 10 mM, pH 7.4), MES buffer (50 mM, pH 5.5), and annealing buffer (10 mM Tris-HCl, 1 mM EDTA, 50 mM NaCl, pH 7.4) were prepared using ultrapure water (Beijing, China, 18.2 MΩ·cm).

Apparatus: TECAN Spark microplate reader (Decan Technology, Ecublens, Switzerland); PB-10 pH meter and Sartorius analytical balance (Sartorius Scientific Instruments, Beijing, China); ST8R high-speed refrigerated centrifuge (Themo Fisher Scientific, Waltham, MA, USA); Panasonic autoclave sterilizer MLS-3751 (Kadoma, Japan); SW-CJ-2FD ultra-clean bench (Suzhou, China), and THZ-98C shaking incubator (Shanghai Shipping Co., Ltd., Shanghai, China).

### 2.2. Preparation of Biosensors

(1) Thiol reduction of substrate chain DNA

The reduction reagent was prepared by dissolving 4.0 mg of tris (2-carboxyethyl) phosphine (TCEP) in 100 µL of PBS buffer (pH 7.4), which was subsequently mixed thoroughly with 100 µL of disulfide-bond-containing substrate chain solution, and the reduction reaction was completed by incubation for 1 h under room temperature conditions. After the reaction, 200 µL of 6% (*v*/*v*) acetic acid, 50 µL of 3 M sodium acetate, and 1.6 mL of anhydrous ethanol were added sequentially for precipitation, and the mixed system was centrifuged at 4 °C for 10 min at 3000× *g*. The supernatant was removed, and the final precipitate was resuspended with 98.2 µL of PBS buffer (10 mM, pH 7.4), to obtain the final concentration of 100 µM of reduced substrate chain solution.

(2) Coupling of Au@Fe_3_O_4_ nanoparticles with substrate chain

A 50 µL aliquot of gold nanomagnetic bead suspension (5 mg/mL) was transferred to a 1.5 mL centrifuge tube. Following magnetic separation and supernatant removal, the beads were washed three times with PBS buffer (pH 7.4) to eliminate unbound impurities. Subsequently, 50 µL of thiol-reduced substrate chain (final concentration 50 µM) was introduced to the bead complex. The substrate chain mixture was subjected to continuous agitation at room temperature for 6 h to facilitate directional conjugation between the substrate chain and the bead surfaces.

(3) Fabrication of DNA nanodendrimer branches

Y-B1, Y-B2, and Y-B3 single-stranded DNA (ssDNA) solutions (initial concentration: 100 µM each) were precisely aliquoted in a 1:1:1 molar ratio into 200 µL thin-walled PCR tubes containing 10 mM Tris-HCl annealing buffer (pH 7.5). The mixture was homogenized using a vortex mixer and subjected to thermal cycling in a PCR instrument. Initial denaturation at 95 °C for 3 min ensured complete dissociation of secondary structures. Subsequently, a programmed cooling phase (0.1 °C/s ramp rate) was implemented to gradually lower the temperature to 25 °C, enabling precise complementary base pairing-driven self-assembly into thermodynamically stable Y-shaped 3D architectures. Post-annealing, samples were immediately stored at 4 °C to minimize structural relaxation.

For L-shaped branches (L1 and L2), no additional annealing was required due to pre-validated thermodynamic stability and structural homogeneity via differential scanning calorimetry (DSC) in prior optimization studies. These components were maintained in their native solution state at −20 °C until further use assembly of Y-shaped DNA nanodendrimers.

(4) Functionalization of Au@Fe_3_O_4_ nanoparticles

Lyophilized DNAzyme-specific strands were centrifuged (10,000 rpm, 2 min) and dissolved in sterile Tris-HCl buffer (pH 7.5) to prepare a 100 µM stock solution. A 50 µL aliquot of diluted DNAzyme solution (50 µM) was incubated with Au@Fe_3_O_4_-sub nanoparticles for 2 h at room temperature. Unbound DNAzyme was removed via magnetic separation, followed by three sequential washes with Tris-HCl buffer (pH 7.5) to eliminate residual impurities. The functionalized nanoparticles were resuspended in the same buffer, yielding the DNAzyme-modified composite probe Au@Fe_3_O_4_-sub-DNAzyme.

Subsequently, an iterative assembly strategy was implemented: dendritic L- and Y-shaped DNA nanostructures were sequentially grafted onto the Au@Fe_3_O_4_ surface using identical thermal annealing protocols. After each assembly step, fluorescence intensity variations on the nanoparticle surfaces were quantitatively monitored using a multi-mode microplate reader (excitation/emission wavelengths: 490/520 nm) to validate layer-by-layer DNA structure integration efficiency.

### 2.3. Gel Electrophoresis Protocol

The agarose gel (0.8% *w*/*v*) was prepared by dissolving agarose powder in 1× TAE buffer (pH 8.3) via intermittent microwave heating, followed by cooling to 55–60 °C, addition of 2 µL Gel Red nucleic acid stain (10,000×), and casting. DNAzyme and substrate chain (3.1 µ mol/L in DEPC-treated water) were hybridized by denaturation at 95 °C for 5 min and gradual cooling (30 min, dark). Annealed DNAzyme/Sub complexes, DNAzyme alone, and substrate chain alone were mixed with 6× DNA loading buffer (bromophenol blue) and loaded onto the gel. Electrophoresis was performed at 150 V for 30 min in 1× TAE buffer until the tracking dye migrated 3/4 of the gel length. Parallel experiments (groups L and Y) were conducted under identical conditions. Post-electrophoresis, DNA bands were visualized using a UV transilluminator and documented with a gel imaging system.

### 2.4. Quantitative Detection of Staphylococcus aureus

The optimized Au@Fe_3_O_4_ nanoparticles (5 mg/mL) were incubated with *S. aureus* suspensions (0, 1 × 10^1^–1 × 10^7^ CFU/mL) in a 25 °C shaking incubator for 10 min. Post-incubation, unbound components were removed via magnetic separation, followed by three washes with PBS (10 mM, pH 7.4, 50 µL each). The Au@Fe_3_O_4_ nanoparticles were resuspended in 50 µL PBS and transferred to a black 96-well plate for fluorescence measurement using a microplate reader.

### 2.5. Specificity Assessment Against Non-Target Bacteria

To evaluate specificity, the biosensor was tested against *Salmonella* spp., *Bacillus subtilis*, *Escherichia coli*, *Streptococcus agalactiae*, and *Proteus* spp. (all at 1 × 10^7^ CFU/mL). Each bacterial suspension underwent identical processing (incubation, magnetic washing, and fluorescence detection) under standardized conditions to ensure comparability.

### 2.6. Real-Sample Analysis in Complex Milk Matrix

To evaluate the biosensor’s performance in complex matrices, pasteurized whole milk was spiked with *S. aureus* (1 × 10^2^ CFU/mL, 1 × 10^4^ CFU/mL, 1 × 10^6^ CFU/mL; *n* = 3 per group) after pretreatment (lipid removal via centrifugation at 10,000× *g*, 4 °C, 15 min; sterilization using a 0.22 µm filter). For detection, 50 µL of optimized Au@Fe_3_O_4_ Au@Fe_3_O_4_ nanoparticles (5 mg/mL) were mixed with 50 µL spiked milk and incubated in a 25 °C shaking incubator (200 rpm, 1 h). Unbound components were removed via magnetic separation (5 min), followed by three washes with PBS (10 mM, pH 7.4, 500 µL each). The Au@Fe_3_O_4_ nanoparticles were resuspended in 50 µL PBS, transferred to a black 96-well plate, and analyzed for fluorescence.

## 3. Results and Discussion

### 3.1. Detection Principle

**Figure 1:**
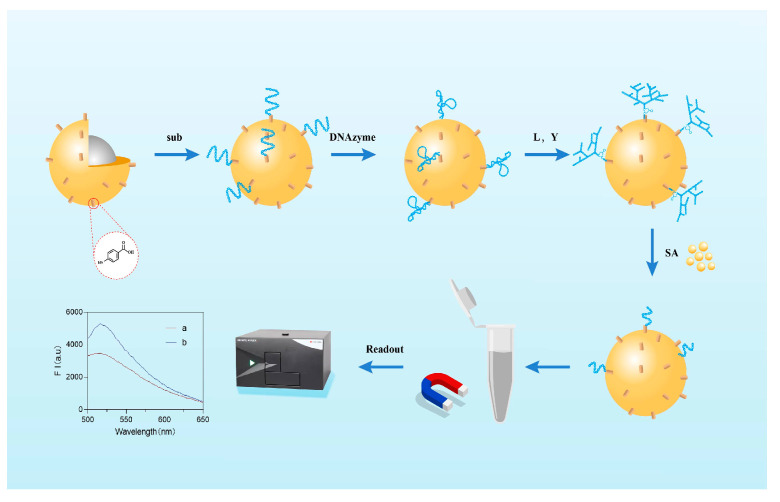
Schematic diagram of fluorescence biosensor detection principle.

The principle of the designed fluorescent biosensor for the detection of *Staphylococcus aureus* is given in Figure 1. A novel ultra-sensitive fluorescent biosensor was prepared by modifying Au@Fe_3_O_4_ using dendritic DNA with fluorescent signals, followed by the addition of L- and Y-type DNA to amplify the fluorescent signals of this sensor. In the presence of *Staphylococcus aureus*, its DNA cleaves the substrate chain at the rA site. In the presence of *S. aureus*, there is only a short substrate chain on the surface of the magnetic bead instead of a complete dendritic DNA, whose fluorescence intensity is diminished. Therefore, the presence of *S. aureus* was determined by detecting the fluorescence intensity of Au@Fe_3_O_4_.

### 3.2. DNA Gel Electrophoresis

**Figure 2:**
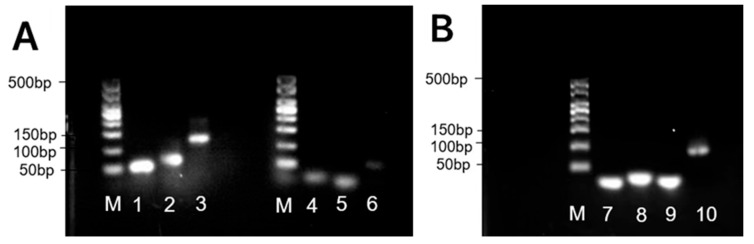
(**A**) M: Marker; 1: substrate chain; 2: DNAzyme; 3: substrate chain + DNAzyme; 4: L1; 5: L2; and 6: L1 + L2. (**B**) 7: Y1; 8: Y2; and 9: Y3; 10: Y1 + Y2 + Y3.

Non-denaturing PAGE analysis confirmed specific binding between the engineered DNA constructs. As shown in Figure 2, hybridization of the DNAzyme with its substrate chain resulted in a stable double-stranded complex, evidenced by significantly retarded band migration (lane 3) compared to individual substrate chain (lane 1) and DNAzyme (lane 2). Similarly, the L1/L2 complementary pair and the Y1/Y2/Y3 single-stranded complex exhibited slower migration than their individual components, confirming complex formation. These results demonstrate the specific binding capability of all designed sequences, providing the essential molecular interaction basis for constructing functionalized DNA nanodendrimers.

### 3.3. Optimization of Conditions

**Figure 3:**
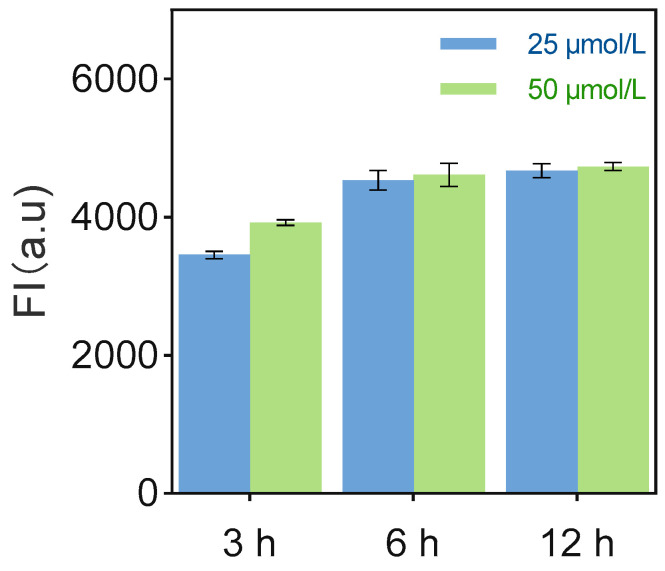
Concentration-time dependent kinetic characterization of substrate chain adsorption on Au@Fe_3_O_4_ surfaces (Each data point was an average of measurements from three independent biosensors).

UV-Vis spectroscopy revealed concentration-independent binding kinetics and surface saturation of substrate chain on Au@Fe_3_O_4_. Analysis at 520 nm (characteristic of the 3′-FAM label on substrate chain) showed no significant fluorescence intensity change for substrate chain alone over 3 h (Figure 3). Upon addition of Au@Fe_3_O_4_, fluorescence intensity significantly increased by 6 h for both substrate chain concentrations (25 µmol/L and 50 µmol/L), indicating binding. No significant further increase occurred at 12 h, demonstrating binding site saturation within 6 h. This saturation kinetics, independent of substrate chain concentration, indicates that the maximum substrate chain loading capacity is determined by the specific surface area and surface sulfhydryl group density of the Au@Fe_3_O_4_ nanomaterial.

### 3.4. Test Certificate

**Figure 4:**
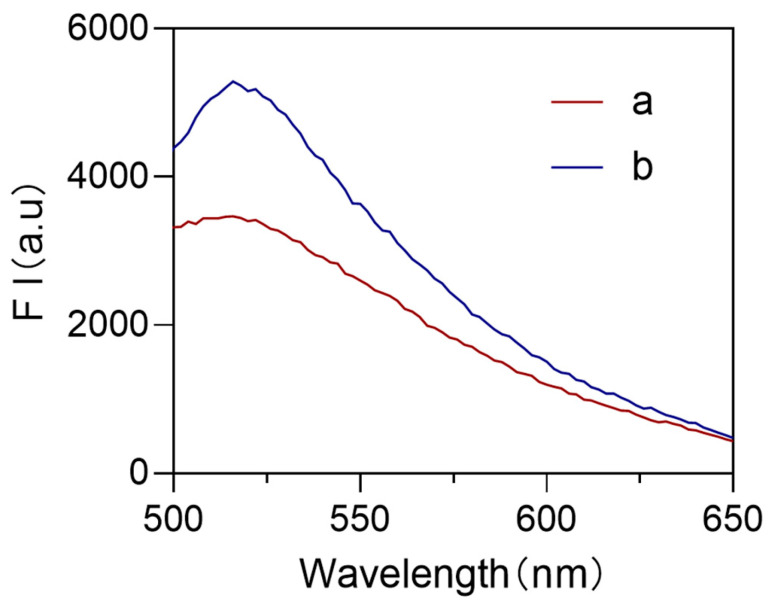
Changes in sensor fluorescence intensity before and after the addition of *Staphylococcus aureus*, a: After addition of *S. aureus*, and b: Before addition.

DNAzyme binds to the magnetic bead-immobilized substrate chain, forming a stable Au-substrate chain-DNAzyme complex. This process is confirmed by a significant increase in fluorescence intensity (Figure 4b, blue curve). When *Staphylococcus aureus* is introduced, its DNA specifically cleaves the rA site in the substrate chain, leading to the dissociation of the complex. This process is confirmed by a significant decrease in fluorescence intensity (Figure 4a, red curve). The results indicate that bacterial DNA induces cleavage of the substrate chain, inactivating the DNAzyme complex. This molecular recognition-based signal response provides a foundation for developing highly sensitive biosensors.

### 3.5. Sensor Performance Optimization

**Figure 5:**
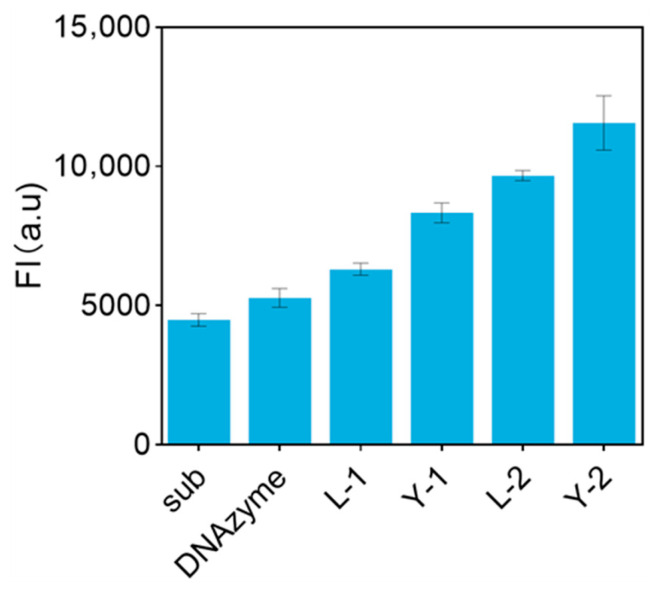
Plot of fluorescence intensity changes in DNA nanodendrimer (Each data point was an average of measurements from three independent biosensors).

In order to investigate the mechanism of multidimensional DNA nanostructures on the amplification of sensing signals, this study constructed a three-dimensional branched DNA nanodendrimer structure by introducing L-type and Y-type DNA units twice, and systematically evaluated its optimization effect on the performance of fluorescent biosensors. The DNA nanodendrimer enhanced the fluorescence intensity 2.8-fold (Figure 5), indicating geometric signal amplification, which confirms that the three-dimensional branching structure can achieve sensor fluorescence intensity gain through the intensive labelling of the FAM fluorescent moieties.

### 3.6. Morphological Characterization of Nanocomplex Assembly

**Figure 6:**
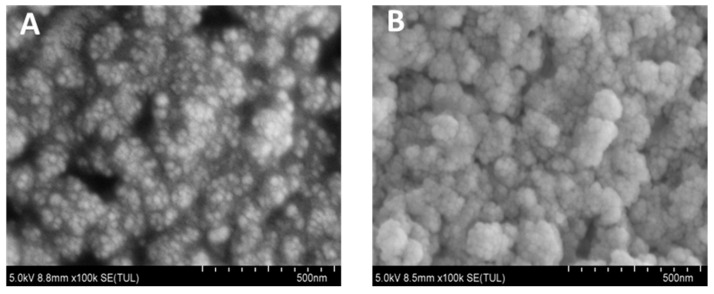
(**A**) SEM image of Au@Fe_3_O_4_; (**B**) SEM image of Au@Fe_3_O_4_-DNA nanodendrimers.

SEM analysis revealed distinct morphological transformations upon nanodendrimer conjugation to Au@Fe_3_O_4_ nanoparticles. Unmodified particles (Figure 6A) exhibited smooth surfaces, while modified counterparts (Figure 6B) showed dendritic protrusions and surface restructuring. These alterations confirm successful immobilization of DNA nanodendrimers on magnetic substrates.

### 3.7. Optimization of Detection Time

**Figure 7:**
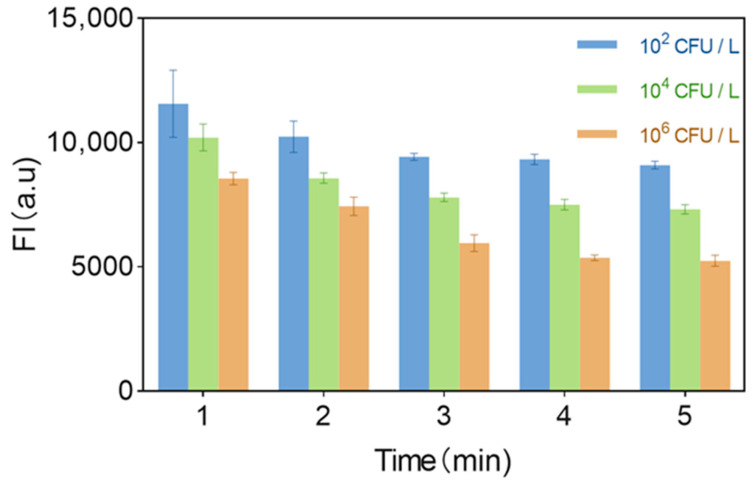
Fluorescent biosensor detects time-dependent fluorescence intensity values of bacteria (each data point was an average of measurements from three independent biosensor).

Detection time optimization revealed fluorescence intensity decreased linearly within 1–5 min during *S. aureus* detection (10^2^–10^6^ CFU/mL; Figure 7). Maximum signal variation occurred at 3 min, establishing this as the optimal detection time to resolve target concentration while maintaining rapid response kinetics. Thus, the optimal detection time is 3 min.

### 3.8. pH Value of Sensor Solution

**Figure 8:**
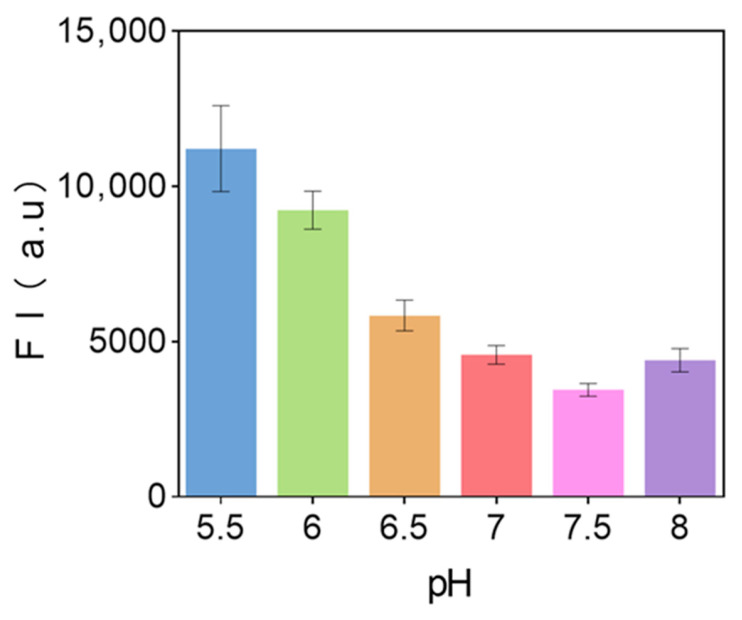
The effect of substrate pH on the performance of fluorescent biosensors (each data point was an average of measurements from three independent biosensors).

pH value is a critical factor influencing the performance of fluorescent biosensors, as it affects the activity of biomaterials as well as the structure and performance of DNAzymes. Under constant conditions, the fluorescence intensity of the substrate solution was measured using 10^7^ CFU/mL *Staphylococcus aureus* at pH values of 5.5, 6.2, 6.8, 7.0, 7.4, and 7.8. The experimental results are shown in Figure 8 As the pH of the substrate increases within the range of 5.5–7.5, the fluorescence intensity decreases. When the pH exceeds 7.4, the fluorescence intensity increases with the increasing pH. Therefore, the optimal pH for the substrate is 7.4.

### 3.9. Stability of Sensor Solution

**Figure 9:**
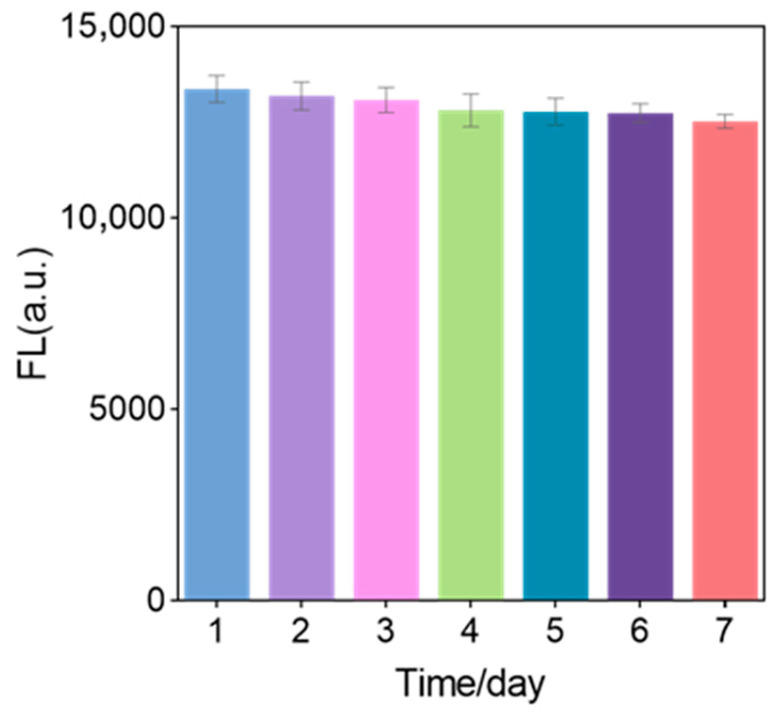
Stability of fluorescent biosensors (each data point was an average of measurements from three independent biosensors).

Expose the sensor to room temperature for 7 days, measuring the fluorescence intensity daily to check the sensor’s stability. As shown in Figure 9 the fluorescence intensity of the sensor after 7 days is 93.5% of the original fluorescence intensity, indicating that the sensor has excellent stability.

### 3.10. Linear Relationship

**Figure 10:**
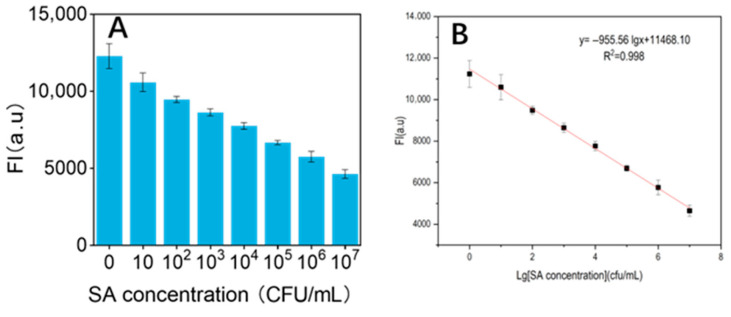
(**A**) Plot of fluorescence intensity of bacteria at different concentrations detected by the fluorescent biosensor; (**B**) Linear plot of *Staphylococcus aureus* detected by the fluorescent biosensor (Each data point was an average of measurements from three independent biosensors).

In this experiment, the detection performance of this biosensor was investigated in the range of *S. aureus* concentration from 1 to 10^7^ CFU/mL under optimized conditions. It can be seen from the Figure that the fluorescence intensity decreases with the increase of *S. aureus* concentration. Figure 10 shows a good linear relationship between the logarithm of *S. aureus* concentration and the fluorescence signal. The linear regression equation is y = −955.56lgx + 11,468.10, where x is the *S. aureus* concentration and y is the fluorescence intensity value. The LOD of the sensor was 1 CFU/mL.

Compared with many reported sensors for detecting *Staphylococcus aureus*, as shown in Table 2, those that rely primarily on fluorescence or electrochemical biosensors have significant advantages. Traditional fluorescence- and electrochemical-based sensors typically have narrow linear ranges and high detection limits. Moreover, and crucially, this biosensor achieves detection within just three minutes, representing the fastest detection time reported among the compared methods. In contrast, the fluorescence biosensing method using DNAzymes offers superior detection performance, with a wider linear range and lower detection limits.

### 3.11. Anti-Interference

**Figure 11:**
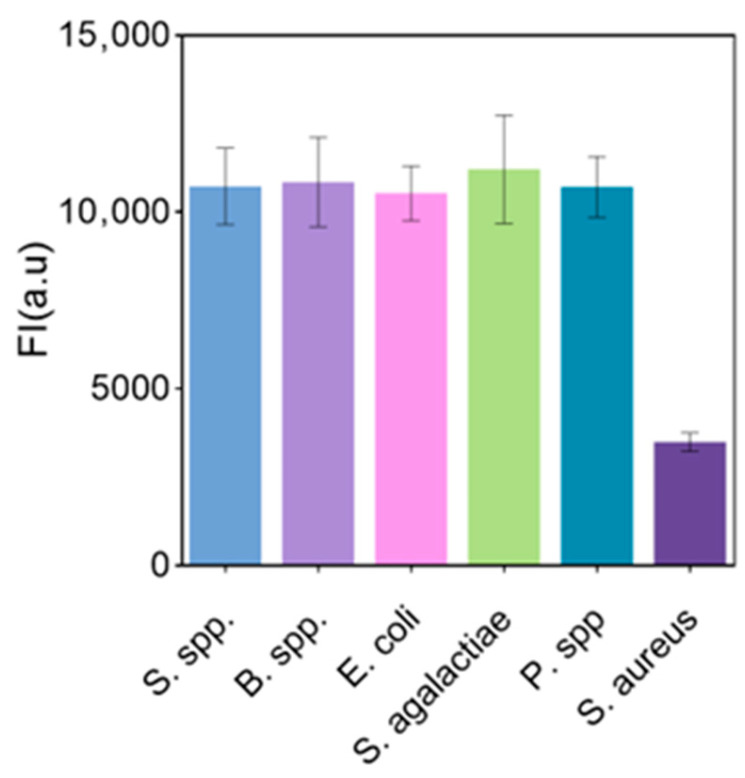
Fluorescence biosensor detects changes in fluorescence intensity of different bacteria (each data point was an average of measurements from three independent biosensors).

To evaluate the selectivity of the sensing system, *Salmonella*, *Bacillus*, *Escherichia coli*, *Streptococcus lactis*-free, and *Proteus* solutions were detected using the sensor. The relative fluorescence intensity of the biosensor for these bacteria was much lower than the response of the aptamer sensor for *S. aureus* at the same concentration, verifying the good specificity of the developed aptamer sensor for the detection of *S. aureus* (Figure 11).

### 3.12. Analysis of the Actual Sample

To investigate the usefulness of the sensor, a known concentration of *Staphylococcus aureus* was added to a milk sample and the sensor was used to measure the bacterial concentration. As shown in Table 3, the recoveries of the biosensor ranged from 85.6–102.1% with a relative standard deviation (RSD) ≤ 8.45%. The experimental results show that the method can be used for the detection and measurement of *S. aureus* in real samples.

## 4. Conclusions

Compared to existing nanotech-based biosensors, this study establishes a novel fluorescent biosensor by integrating DNAzyme-functionalized Au@Fe_3_O_4_ nanoparticles (immobilized via Au–S bonds) and signal-amplifying Y-dendrimers for ultrasensitive *Staphylococcus aureus* detection. The target-activated DNAzyme cleavage releases fluorescent dendrimers, enabling quantifiable signal reduction within 3 min. The platform achieves an exceptional detection limit (1 CFU/mL), broad linearity (1–10^7^ CFU/mL), high specificity against competing pathogens, and demonstrated practical utility (recoveries: 85.6–102.1%). This work provides a rapid, field-deployable strategy for on-site monitoring of dairy contaminants, advancing DNA nanotechnology in food safety diagnostics. However, the long-term stability of the fluorescent nanobranch-functionalized Au@MBS nanocomposite still needs to be optimized.

## Figures and Tables

**Table 1:** DNA oligonucleotides used in this study.

Name	Sequence and Modifications (From 5′–3′)
RL-Sub-SH-HS	CTAATGAGTACCTACTGTCTCTGGATGATCCTATGAACTGACT/rA/TGACCTCACTACCAAGGCGCTATCGGGAAG-FAM
RL-DNAzymes	FAM-ATGCCATCCTACCAACCACGAAGTACATTTCAAACTCATAACAATCCATCGGTTAGGTCCT GGTTGGAGCTCTGAACTCGAGACAGTAGGTACTCATTAG-FAM
Y-1	FAM-CACGCAGAGTAACACATGACCGTCGAAGCTTCCCGATAGCGC-FAM
Y-2	FAM-CTTCGACGGTCATGTACTAGATCAGAGGCTTCCCGATAGCGC-FAM
Y-3	FAM-CCTCTGATCTAGTATGTTACTCTGCGTGCTTCCCGATAGCGC-FAM
L1	FAM-TCTATTCGCATGAGAAGCGCTATCGGGAAG-FAM
L2	FAM-TTCTCATGCGAATAGAGCGCTATCGGGAAG-FAM

**Table 2:** Compare the detecting performances of DNA nanodendrimer-based fluorescent biosensor with other sensors.

Sensor	Method	Linear Range (CFU/mL)	Detection Limit (CFU/mL)	Testing Time (min)	Reference
RPA-CRISPR/Cas12a-Eu-MOF	Fluorescence	7.9 × 10^0^–7.9 × 10^8^	3	-	[43]
Aptamer-based Fluorescent LFB	Fluorescence	2.8 × 10^1^–2.8 × 10^7^	1.65	-	[44]
AuNSs@PB@Ag-Apt sensor	Colorimetric probe	1 × 10^0^–1 × 10^8^	1	-	[45]
ECL Biosensor	Electrochemi-luminescence	1 × 10^1^–1 × 10^9^	1.16	40	[46]
Aptamer-Modified GaN HEMT Sensor	GaN HEMT	1 × 10^2^–1 × 10^7^	19	3.33	[47]
AuNRs–BICC Click Chemistry Biosensor	click chemistry	1 × 10^1^–1 × 10^7^	10	180	[48]
DNA nanodendrimer-based fluorescent biosensor	Fluorescence	1–1 × 10^7^	1	3	This work

**Table 3:** Recovery results of the fluorometric biosensor in milk samples containing *S. aureus* against the plate counting method.

Samples	Samples (CFU/mL)	Plate Counting Method ± SD (CFU/mL)	Fluorescent Biosensing ± SD (CFU/mL)	RSD (%)	Recovery (%)
Milk-1	1 × 10^2^	(0.983 ± 0.064) × 10^2^	(0.967 ± 0.053) × 10^2^	5.48	98.4
	1 × 10^4^	(0.991 ± 0.058) × 10^4^	(1.012 ± 0.065) × 10^4^	6.18	102.1
	1 × 10^6^	(1.061 ± 0.069) × 10^6^	(1.003 ± 0.057) × 10^6^	5.34	94.5
Milk-2	1 × 10^2^	(1.045 ± 0.051) × 10^2^	(0.895 ± 0.043) × 10^2^	4.80	85.6
	1 × 10^4^	(1.026 ± 0.064) × 10^4^	(0.911 ± 0.048) × 10^4^	5.27	88.8
	1 × 10^6^	(1.071 ± 0.035) × 10^6^	(1.006 ± 0.085) × 10^6^	8.45	93.9
Milk-3	1 × 10^2^	(1.063 ± 0.052) × 10^2^	(0.987 ± 0.068) × 10^2^	6.89	92.9
	1 × 10^4^	(0.995 ± 0.047) × 10^4^	(0.965 ± 0.049) × 10^4^	5.08	95.5
	1 × 10^6^	(1.039 ± 0.062) × 10^6^	(0.983 ± 0.069) × 10^6^	7.02	94.6

## Data Availability

Data are contained within the article.
